# Ecotoxicological impact of mosquitocidal jasmine-based oils nanoemulsion on Nile tilapia fish *Oreochromis niloticus*

**DOI:** 10.1038/s41598-025-25440-3

**Published:** 2025-11-18

**Authors:** Reham E. Muhammed, M. A. Al-Zahaby, Gamila A. M. Kotb, Salwa M. Abdallah, Hanan A. A. Taie, Ahmed A. Gh. Farag, Hanem F. Khater, Ibrahim Taha Radwan, Nashwa Elshaer

**Affiliations:** 1 Center of Excellence for Toxicological Testing, Mammalian and Aquatic Toxicology Dept., Central Agricultural Pesticides Laboratory, Agricultural Research Center, Dokki, 12618 Giza Egypt; 2 Central Laboratory for Aquaculture Research, Hatchery and Fish Physiology Department, Agricultural Research Center, Abbassa, Egypt; 3 Plant Biochemistry Department, Agricultural and Biological Research Institute, National Research Centre, 33 El-Bohouth St. (Former El-Tahrir St.), Dokki, 12622 Cairo Egypt; 4 Plant Protection Department, Agriculture Faculty, Zagazig University, Zagazig, 44511 Egypt; 5 Parasitology Department, Faculty of Veterinary Medicine, Benha University, Toukh, 13736 Egypt; 6 Supplementary General Sciences Department, Faculty of Oral and Dental Medicine, Future University in Egypt, Cairo, 11835 Egypt

**Keywords:** Jasmine oil nanoemulsion, Nile Tilapia, Eco-friendly pesticide, Oxidative stress, Histopathology, Biochemical marker, Biological techniques, Health care

## Abstract

Nanoemulsion technology has become a promising approach for improving stability and bioavailability of natural products, providing more efficient and eco-friendly biocides. Jasmine oil, derived from *Jasminum spp.*, represents a natural product with mosquitocidal properties. This study investigated the potential pathophysiological effects of jasmine oil nanoemulsion (JA-NE) on Nile tilapia (*Oreochromis niloticus*), a species of economic importance in Egypt. The acute toxicity assessment indicated a 96-h LC₅₀ value above 100 mg/L, with no adverse effects observed at sublethal doses. However, chronic exposure at sublethal concentrations of 5 and 10 mg/L (5 and 10%) for 28 days revealed significant elevation in hepatic enzymes (AST, ALT) and creatinine, indicating potential liver and kidney stress. Lipid peroxidation and antioxidant responses were also emphasized by a significant increase in oxidative stress biomarkers, particularly malondialdehyde (MDA), and mixed responses of glutathione (GSH) and glutathione-S-transferase (GST) levels. Additionally, immune functions were altered where proinflammatory cytokines, interleukin-1β (IL-1β) and tumor necrosis factor (TNF-α), exhibited concentration-dependent changes. Histopathological analysis revealed tissue damage in gills, liver, kidneys, and brain, showing lamellar fusion, hepatocyte vacuolation, and renal tubular necrosis. In conclusion, although JA-NE exhibited low acute toxicity, but its prolonged exposure to sublethal doses can induce physiological risks to aquatic organisms. Such findings underscore the need for careful consideration of dosage and exposure duration of nanoemulsions to balance their benefits versus their potential aquatic ecosystem impacts.

## Introduction

The escalating global demand for sustainable agricultural strategies has spurred concern in eco-friendly alternatives to conventional pesticides, which are often involved in negative impacts on non-target organisms and ecosystems^1–3^. Plant-based pesticides, such as essential oils, have drawn a lot of interest among these alternatives owing to their low toxicity, biodegradability, and minimal environmental persistence^4–7^.

Jasmine oil, which is extracted from *Jasminum* spp. and renowned for its aromatic scent, is one such natural substance that has antifungal, antibacterial, and insecticidal qualities^8,9^. Its essential oils have demonstrated larvicidal and antibacterial properties against a range of pests and diseases, indicating its availability in aquaculture^10,11^. However, the application of essential oils in agriculture is often limited by their low water solubility, volatility, bioavailability, and instability^1,9^.

To overcome these challenges, nanotechnology has emerged as a viable strategy to enhance the bioavailability, stability, and delivery of essential oils. However, consideration must be given to the environmental safety of these botanical pesticides, especially when prepared in delivery systems like nanoemulsions. Nanoparticles, which are nanoscale dispersions of oil in water, have emerged as a promising delivery system for bioactive compounds, including essential oils^12,13^.

They are a desirable alternative for boosting the effectiveness of ecofriendly insecticides due to their small droplet size, stability, and increased bioavailability^14^. Jasmine oil, when formulated as a nanoemulsion, may provide a more effective and safe method for controlling pests^10^, reducing the dosage needed and lowering any ecological hazards. Jasmine oil nanoemulsions have been shown in studies to have promising larvicidal efficacy against pests such as *Culex pipiens*, which makes them eco-friendly pest control candidates^10^. Studies on nanoemulsions of botanical oils such as neem, clove, citrus, and thyme have been evaluated for both pesticide efficacy and safety in aquatic organisms. Previous studies on neem oil nanoemulsions showed enhanced bioavailability, antimicrobial activity, and altered toxicity profiles compared to bulk formulations.

Nanomaterials were released into the environment, particularly the aquatic environment, due to their extensive use. Aside from their many advantages, nanomaterials have recently become a double-edged weapon that requires research to determine their potential toxicity to living things^15,16^. Therefore, evaluating the ecotoxicity of botanical pesticide-containing nanoemulsions is essential to guaranteeing their safe and long-term use.

Since aquatic organisms are frequently the first to be impacted by contaminants, assessing the safety and sustainability of essential oil nanoemulsion requires an understanding of how it affects fish health^17,18^. Nile tilapia (*Oreochromis niloticus*) is a highly valuable fish species extensively farmed in aquaculture systems worldwide, with significant ecological relevance to freshwater habitats^19^. Tilapia is a perfect model organism for evaluating the possible environmental effects of jasmine oil nanoemulsion since it offers important insights into the possible effects of agrochemicals on aquatic life. Comparative toxicological assessments have shown that *O. niloticus* often exhibits heightened sensitivity to environmental pollutants compared to other freshwater species such as *Labeo rohita*. For instance, under exposure to heavy metals like cadmium and chromium, *O. niloticus* demonstrated more significant declines in antioxidant enzyme activity and more severe tissue alterations than *L. rohita*^20–22^. Furthermore, consistent vulnerability of tilapia to pesticides and nanoparticle exposure supports its role as a sentinel species in aquatic toxicity evaluation^23^. These characteristics justify its selection in the current study to assess the sub lethal effects of JA-NE exposure on oxidative biomarkers and tissue integrity.

The study will investigate critical biomarkers related to oxidative stress, immune function, and tissue integrity, such as liver, gill, kidney, and brain. The findings will contribute to the growing body of knowledge on sustainable pest management strategies and inform the development of safer, more environmentally benign alternatives to conventional chemical pesticides.

## Materials and methods

### Plant material

The essential oils of jasmine oil blend (*Jasminum sambac* L., and *Jasminum azoricum* L.) were purchased from Cap Pharm’s EL CAPTAIN Company, a supplier specializing in essential oils and cosmetics located in El Obour City, Cairo, Egypt.

### Jasmine oil phytochemical analysis

The chemical composition of jasmine essential oil (JA) was previously determined by gas chromatography–mass spectrometry (GC–MS) in a study by Radwan^10^, where nineteen compounds were identified, predominantly monoterpenes and esters. The major constituents included linalyl acetate (6.50%), 4-aminohydrazide benzoic acid (3.19%), and 2-(dimethylamino)-5, 6-dimethyl-1H-pyrimidin-4-one (2.38%).

### Synthesis of jasmine oil blend oil/water nanoemulsion

The synthesis of jasmine oil blend nanoemulsion, homogenization method was used with little modifications according to Radwan and co-worker^10,13,24^ as follows: in a50-mL beaker (B1) 2.5 g of the JA essential oil was warmed using water bath (40–45 °C. Another beaker (B2) contained 10 mL of distilled water used to dissolve 50 mg of sodium glycocholate, 50 mg of sodium taurocholate, 0.5 mL of butanol (co-surfactant) and 5.5 g of tween 20 were mixed and warmed at the same temperature. For oil–water nanoemulsion preparation the two beakers mixed and stirred very well, finally sonicated for 15 min under cold conditions. The final emulsion was placed in 50 mL-Falcon tube and kept under cold conditions.

### Particle size and the surface charge

The quality of the made nanoparticles was checked by looking at their size and how varied they are using dynamic light scattering (DLS) at a 173° angle at room temperature. Their zeta potential, which helps understand how stable the particles are by measuring changes in scattered light due to their charge at a 12° angle, was also tested. Their zeta potential, which provides important information about colloidal stability by detecting shifts in scattered light caused by particle charge at a 12° angle, was also evaluated. All measurements, including zeta potential, PDI, and particle size, were carried out at the Egyptian Petroleum Research Institute using a Zeta Silver Nano Series (HT) Nano ZS instrument (Malvern Instruments, UK). Before testing, 5–10 mg of each nano formulation was mixed in 10 mL of distilled water using a sonication bath and then placed into a quartz cuvette. Three independent readings were recorded for both the PDI and size distribution, and the most representative values were reported.

### Surface morphology estimation by transmission electron microscope (TEM)

Transmission electron microscopy (TEM) was used to look at the inside of the NLCS nanoparticles, showing that they have a consistent shape and that how they stick together is important for their stability. High-resolution TEM (HR-TEM) analysis was conducted at the Egyptian Petroleum Research Institute (EPRI) in Cairo using the JSM-7100F system. Imaging was performed with a JOEL Jem-2100-115 HR-TEM operating at an accelerating voltage of 200 kV. To prepare the samples, about 1 µl of NLC nanoparticles was mixed with 200 times more double-distilled water and put on a carbon-coated 200-mesh grid for about 2 min. Excess fluid was removed using filter paper. Next, we added two drops of 2% (w/w) phosphotungstic acid (PTA) for 10 s to negatively stain the grid. We removed the surplus PTA with filter paper, mounted the grid onto the sample holder, and configured the TEM for imaging.

### Ecotoxicity studies

Ecotoxicity studies were carried out on Nile tilapia fish using a 50% nanoemulsion of jasmine oil blend (JA-NE) to assess both short-term and long-term (acute and chronic toxicity) studies.

### Experimental fish

One hundred Nile Tilapia fish (*O. niloticus*) were used in this experiment based on several factors, including their year-round availability, ease of care, and testing convenience. Fish were provided by a fish farm in Ismailia Governorate, Egypt. Their size range was between 6 and 9 cm (40–50 gm). The fish ought to be healthy and devoid of any obvious information (12–16 h. photoperiod daily, 25 °C ± 2). Prior to usage, all fish must be kept in glass tanks with a 100 L capacity (50 × 50 × 50 cm) and exposed to the quality water to be used in the experiment for at least 14 days (dechlorinated if needed). Any disturbances that may change the behavior of the fish needed to be avoided. According to APHA^25^, every aquarium was equipped with an aeration system had identical physicochemical water conditions, including a temperature of 25 ± 2 °C, dissolved oxygen levels of 7–9 mg/L, pH between 7.5 and 8.0, and total hardness of 210 mg/L. Fish were fed daily with an amount equivalent to 3% of their body weight.

### Acute toxicity test

For determination of LC_50_ after 96 h according to OECD No. 203^26^, a range-finding test was performed firstly to identify the appropriate concentration range for the definitive main test using a static system with suitable temperature, dissolved oxygen, and pH. Test concentrations were estimated as 0, 29.63, 44.44, 66.67 and 100 mg/L. A minimum of ten healthy fish were used for each test concentration and examined during the initial 2–4 h and then on a daily basis. Visible abnormalities, including impaired equilibrium, altered swimming behavior, respiratory disturbances, pigmentation changes, morbidity, and mortality, were all recorded. A fish is considered dead when there is no visible movement of the gills and the caudal peduncle fails to respond to tactile stimulation.

### Chronic toxicity test

The environmental negative effects of sublethal concentrations were investigated by using 5% (5 mg/L) and 10% (10 mg/L) of the LC_50_ value as Low (LC) and High Concentration (HC) respectively for 28 days. The experiment was conducted in accordance with OECD Test Guideline No. 204^27^. Chronic exposure of tested nanoemulsion oils on oxidative and immunity biomarkers after the exposure period. Following the experimental period, fish were anesthetized with clove oil at a concentration of 7.4 ml/L, as described by Rezende^28^. Blood was drawn from the caudal vein, centrifuged at 3000 rpm for 10 min, and the serum was carefully separated, collected, and stored at − 80 ° for biochemical and immunological assessment. To gather the various organ tissues, the fish were anesthetized and then euthanized by spinal cord severing. To further elucidate the connection between oxidative stress and tissue damage, this study also identified lipid profiles and hepato-renal toxicity. As well as the histopathological lesions in some vital organs (gills, liver, kidney, and brain), were examined.

### Biochemical analysis

Kits used to measure the serum biomarker analysis of kidney and liver functioning as well as lipid profiles were purchased from Biodiagnostic Company. Ref.^29,30^ methods were used to measure the activity of transaminases aspartate aminotransferase (AST), alanine aminotransferase (ALT), and alkaline phosphatase (ALP). The levels of albumin (Alb) and total protein (TP) were measured using the techniques of^31,32^. Furthermore, the albumin/globulin ratio (A/G ratio), globulin (Glb) level, and ALT/AST ratio were calculated. The urea level was analyzed using the^33^ protocol, and the creatinine concentration was analyzed following the kinetic approach of^34^. Regarding the lipid profiles, the described methods by^35^ were used to measure total lipid level (TL),^36^ for total cholesterol level (TC),^37^ for low-density lipoprotein level (LDL) and^38^ for high-density lipoprotein level (HDL).

### Oxidative stress indicators determination

A 10% (W/V) homogenate was obtained by homogenizing liver and kidney tissues in ice-cold sodium phosphate buffer (50 mM, pH 7) with EDTA (0.1 mM). To evaluate oxidative stress and antioxidant enzyme activity, the homogenates underwent centrifugation at 12,000 rpm for 30 min at 4 °C. The resulting supernatants were then separated and kept at − 40 °C. Malondialdehyde (MDA) concentration was measured using the^39^ method. The techniques of^40^ were used to measure glutathione-s-transferees (GST) level. The content of total glutathione (GSH) was investigated by^41^. Interleukin 1β (IL-1β, Cat. No. EA0049FI) and tumor necrosis factor α (TNF-α, Cat. No. EA0023FI) that were provided by Bioassay Technology Laboratory (BT Lab, www.bt.laboratory.com, China) were used for the assay by using the ELISA apparatus (Chromate 4300, Awareness Technology, China).

### Histopathological examination

Histopathological analysis of the gills, liver, kidney, and brain was carried out in the histopathological unit, histopathology Department. Animal Health Research Institute (AHRI), Agricultural Research Center (ARC), according to^42^. Fish organ samples were fixed in 10% neutral buffered formalin (pH 7.2) and then dehydrated through a sequential ethanol gradient. After clearing with methyl benzoate, the samples were embedded in paraffin wax and sectioned to a thickness of 4–7 μm. After deparaffinization with xylene, Haematoxylin and Eosin (H&E) staining was applied to the sections to enable histological analysis. This procedure was conducted following the method of^43^, as modified by^44^. An Olympus BX51 light microscope (Tokyo, Japan) with an attached digital camera (Olympus C-5050, Olympus Optical Co. Ltd., Japan) was used to analyze and capture images of the sections.

### Data analysis

The LC_50_ (median lethal concentration) of the test item was calculated using the method outlined by^45^. The data (M ± S.E.) were statistically analyzed using one-way ANOVA, with Duncan’s multiple range (at *P* ≤ 0.05, 0.01 and 0.001). All data were analyzed with PASW Statistics software (SPSS version 25).

### Ethical statement and animal welfare

All experiment protocols followed the “Guide for the Care and Use of Laboratory Animals” in accordance and compliance with ARRIVE guidelines (https://arriveguidelines.org) for the use and welfare of experimental animals.

### Ethical approval

The Ethical Committee of Benha University’s Institutional Animal Care (Benha-IACUC), Moshtohor, Benha, Sharkia, Egypt approved all experimental procedures (Approval Number: BUFVTM 01-10-22).

## Results

### Acute toxicity of JA-NE

Both the control and JA-NE-exposed groups exhibited neither feeding behavior changes nor tilapia mortality, as illustrated in Table 1. After 96 h of exposure, the median lethal concentration (LC_50_) values for JA-NE (50% of nanoemulsion (50% NE)) in toxicity studies was over 100 mg/L.

**Table 1:** Values of Acute LC_50_-96 h of JA-NE (50% NE) and its confidence limits (95%) on Nile tilapia (*Oreochromis niloticus*).

Conc. (mg/L)	No. dead	Mortality (%)	LC_50_ value (mg/L)	Confidence limits (95%)	
				Lower	Upper
0	0/5	0	Over 100 mg/ L	–	–
29.63	0/5	0			
44.44	0/5	0			
66.66	0/5	0			
100	0/5	0			

### Biochemical parameters of JA-NE

In the current study, the effects of sublethal concentrations (LC and HC) of JA-NE on the hepatic changes in Nile tilapia fish were assessed after 28 days of exposure. Exposure to both LC and HC of JA-NE resulted in minimal changes in ALT and ALP, TP, Alb, and Glb activities. No significant changes were observed in ALT and ALP levels across the treatment groups. However, a significant increase in AST levels was observed in the HC group (*P* ≤ 0.01), with levels rising from 19.23 ± 0.89 U/L in the control group to 23.57 ± 1.04 U/L (22.57% change), as detailed in Table 2. The ALT/AST ratio was significantly elevated in the LC group (*P* ≤ 0.001), increasing from 0.61 ± 0.04 in the control group to 1.02 ± 0.06.

**Table 2:** Effect of JA-NE (50% NE) on liver functions of Nile tilapia* (Oreochromis niloticus)* for 28 days.

Parameters	Cont	LC	HC
ALT (U/L)	11.62 ± 0.32	11.05 ± 0.31	11.95 ± 0.53
AST (U/L)	19.23 ± 0.89	20.35 ± 0.87	23.57 ± 1.04**
ALT/AST	0.61 ± 0.04	1.02 ± 0.06***	0.51 ± 0.03
ALP (U/L)	42.19 ± 1.38	42.02 ± 0.33	44.48 ± 1.15
TP (g/dL)	4.04 ± 0.07	3.77 ± 0.09	3.77 ± 0.16
Alb (g/dL)	2.79 ± 0.03	2.65 ± 0.09	2.67 ± 0.14
Glb (g/dL)	1.26 ± 0.08	1.12 ± 0.07	1.1 ± 0.07
A/G	2.26 ± 0.14	2.41 ± 0.23	2.46 ± 0.19

**p ≤ 0.01, ***p ≤ 0.001.

**Fig. 1:**
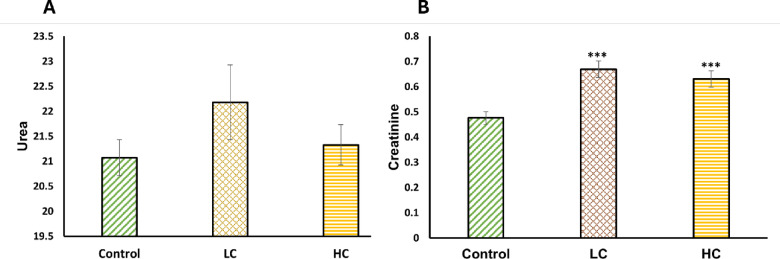
Effect of Jasmine oil nanoemulsion (50% NE) on renal functions of Nile tilapia* (Oreochromis niloticus)* for 28 days. Data are presented as mean ± SE (n = 5). *** significant at *P* ≤ 0.001. Control group, where fish are the control reference without any exposure. LC, Fish Exposed to Low Concentration (5%) of JA-NE; HC, Fish Exposed to High Concentration (10%) of JA-NE.

With respect to renal function, creatinine level was significantly increased in LC and HC of JA-NE groups compared to the control group (*P* ≤ 0.001). The control group showed a creatinine level of 0.48 ± 0.02 mg/dl, while the LC and HC groups exhibited elevated levels of 0.67 ± 0.03 mg/dl and 0.63 ± 0.03 mg/dl, respectively. In contrast, urea levels remained consistent and unaffected in all groups, which did not show any statistically significant changes across the groups compared to the control (Fig. 1).

The lipid profiles, including TL, TC, HDL, and LDL, revealed no discernible alterations in LC of the JA-NE group, as indicated in Table 3. In contrast, fish exposed to the HC of JA-NE exhibited a significant rise (*P* ≤ 0.01) in TC, HDL, and LDL levels compared to the control group, as outlined in Table 3.

**Table 3:** Effect of JA-NE (50% NE) on lipid profile of Nile tilapia* (Oreochromis niloticus)* for 28 days .

Parameters	Cont	LC	HC
T. Lipid (mg/dL)	518.6 ± 21.00	532.5 ± 25.55	535.6 ± 26.63
T. Cholesterol (mg/dL)	106.9 ± 5.63	108.9 ± 2.02	131.5 ± 2.88**
HDL (mg/dL)	50.82 ± 2.05	63.97 ± 2.38*	67.42 ± 4.79**
LDL (mg/dL)	91.14 ± 5.69	89.69 ± 1.84	111.5 ± 3.29**
Risk Ratio	2.12 ± 0.17	1.71 ± 0.09	1.98 ± 0.12

Risk Ratio = T. Cholesterol/HDL.

*p ≤ 0.05, **p ≤ 0.01.

### Oxidative stress biomarkers

**Fig. 2:**
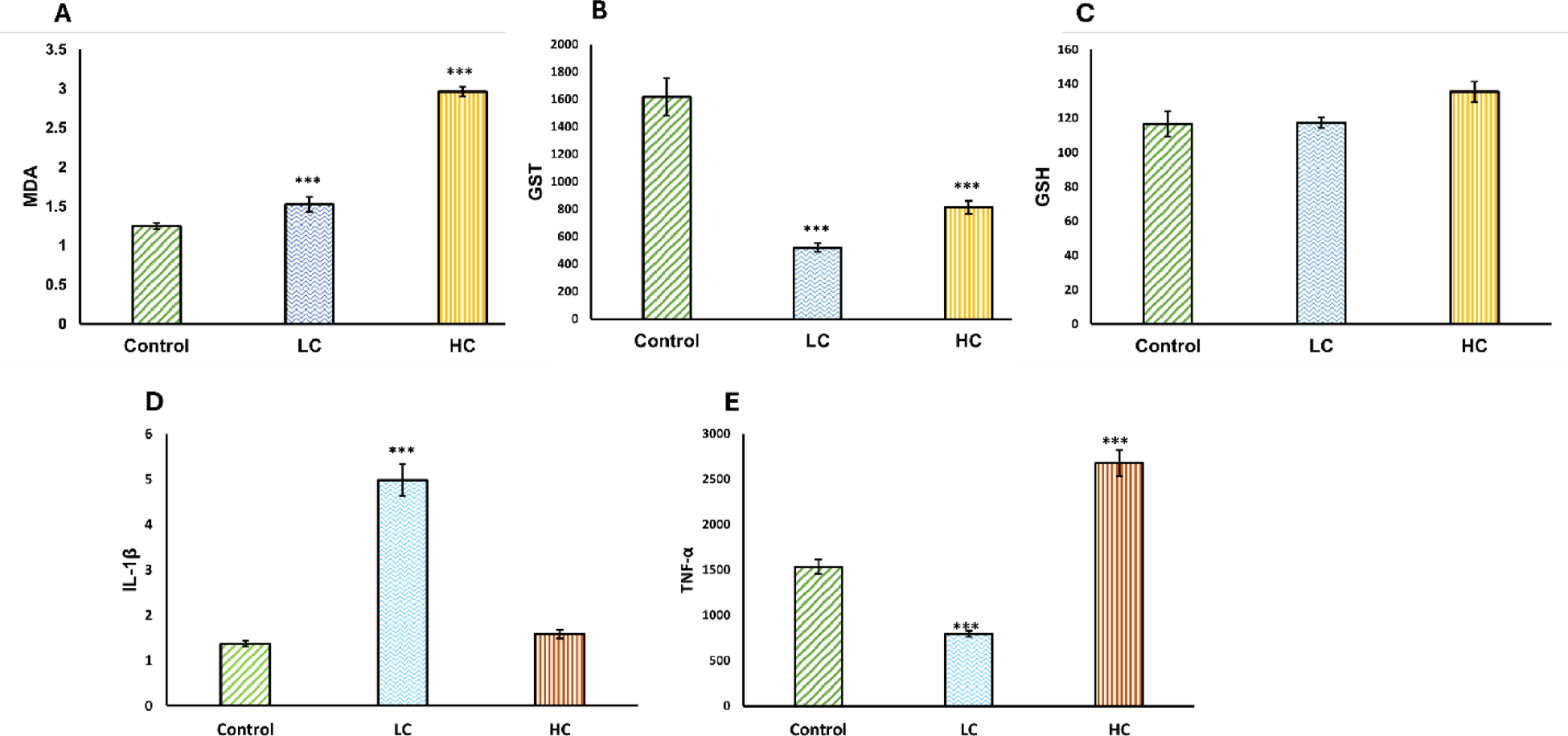
Effect of Jasmine oil nanoemulsion on Serum Oxidative Stress and Antioxidants Biomarkers (**A**, **B**, **C**) and immunological markers (**D**, **E**) of Nile tilapia* (Oreochromis niloticus)* for 28 days. Data are presented as mean ± SE (n = 5). *** Significant at *P* ≤ 0.001. MDA, Malondialdehyde; GSH, Total glutathione; GST, Glutathione-s–transferees; IL-1β, Interleukin-1β; TNF-α, Tumor Necrosis Factor α.

In the present study, exposure to JA-NE (LC and HC) in fish was assessed through the antioxidant response, characterized by the activities of GSH, GST, and MDA enzymes. A significant induction of MDA levels was observed in fish exposed to both LC and HC of JA-NE (*P* ≤ 0.001). The LC and HC groups showed elevated levels of 1.52 ± 0.097 nmol/mL and 2.96 ± 0.058 nmol/mL, respectively, in comparison with the control group, which had an MDA level of 1.25 ± 0.04 nmol/mL (Fig. 2A).

GSH levels showed a slight increase in the JA-NE-exposed groups compared to the control, but this increase was not statistically significant (*P* ≤ 0.05). In contrast to the control group, which had GSH levels of 116.7 ± 7.34 nmol/mL, the LC and HC groups recorded levels of 117.5 ± 3.17 nmol/mL and 135.4 ± 5.88 nmol/mL, respectively (Fig. 2B). A significant reduction in GST activity was observed in fish exposed to both LC and HC of JA-NE (*P* ≤ 0.001). The GST activity of the LC and HC groups was 520.2 ± 31.95 µmol/min/mg protein and 811.6 ± 47.33 µmol/min/mg protein, respectively, whereas the control group’s activity was 1616.7 ± 135.1 µmol/min/mg protein (Fig. 2C).

### Immune responses

To study the effect of JA-NE on the immunotoxicity of Nile tilapia, the proinflammatory cytokines such as IL-1β and TNF-α were assessed (Fig. 2D&E). Regarding the IL-1β level, fish exposed to JA-NE showed a significant activation with LC (4.98 ± 0.35 ng/L) and a slight response of activation with the HC group (1.58 ± 0.1 ng/L) when compared to the control (1.37 ± 0.06 ng/L) (Fig. 2D).

When it comes to TNF-α level, The LC treatment group showed a significant decrease in TNF-α levels (*P* ≤ 0.001), with levels dropping from 1533 ± 82.00 ng/L in the control group to 793.4 ± 30.89 ng/L. Conversely, the HC treatment group exhibited a dramatic escalation in TNF-α levels, reaching 2679 ± 140.5 ng/L (*P* ≤ 0.001) (Fig. 2E).

### Histopathology findings

**Fig. 3:**
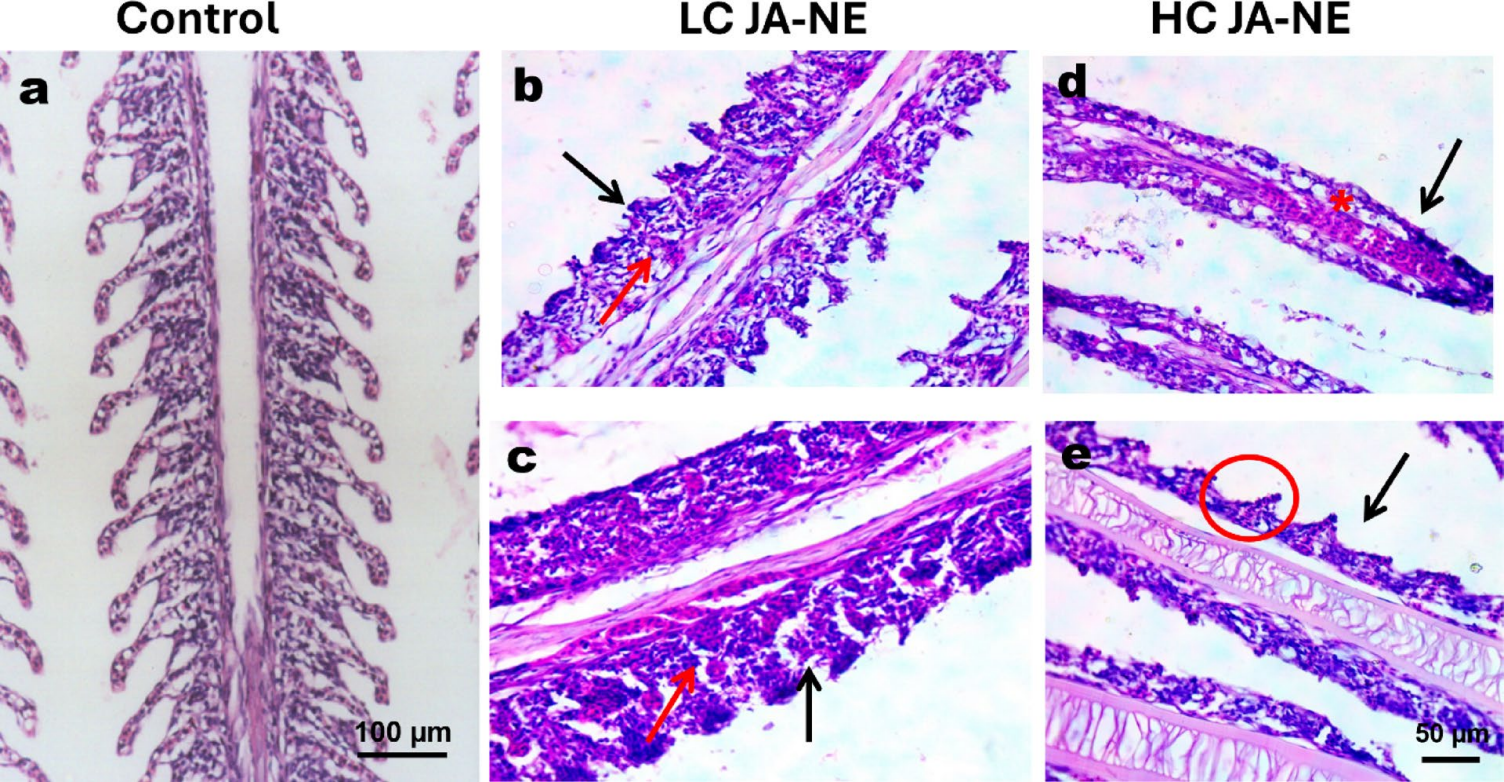
Photomicrograph of histological sections on the gills on Nile tilapia (*Oreochromis niloticus*). (**a**) Control group was conducted without any substance. (**b**, **c**) the gills of *Oreochromis niloticus* exposed to a low concentration (5%) of JA-NE showing partial fusion of secondary gill lamellae with infiltration by mononuclear inflammatory cells infiltration (black arrow), epithelial cell hyperplasia (red arrow). (**d**, **e**) the gills of *Oreochromis niloticus* exposed to a high concentration (10%) of JA-NE showing complete fusion of secondary gill lamellae (black arrow), congestion of blood vessels (red asterisk), epithelial lifting (red circle). Hematoxylin and Eosin stain, scale bar: **a** (100 µm), **b**–**e** (50 µm) (X400).

**Fig. 4:**
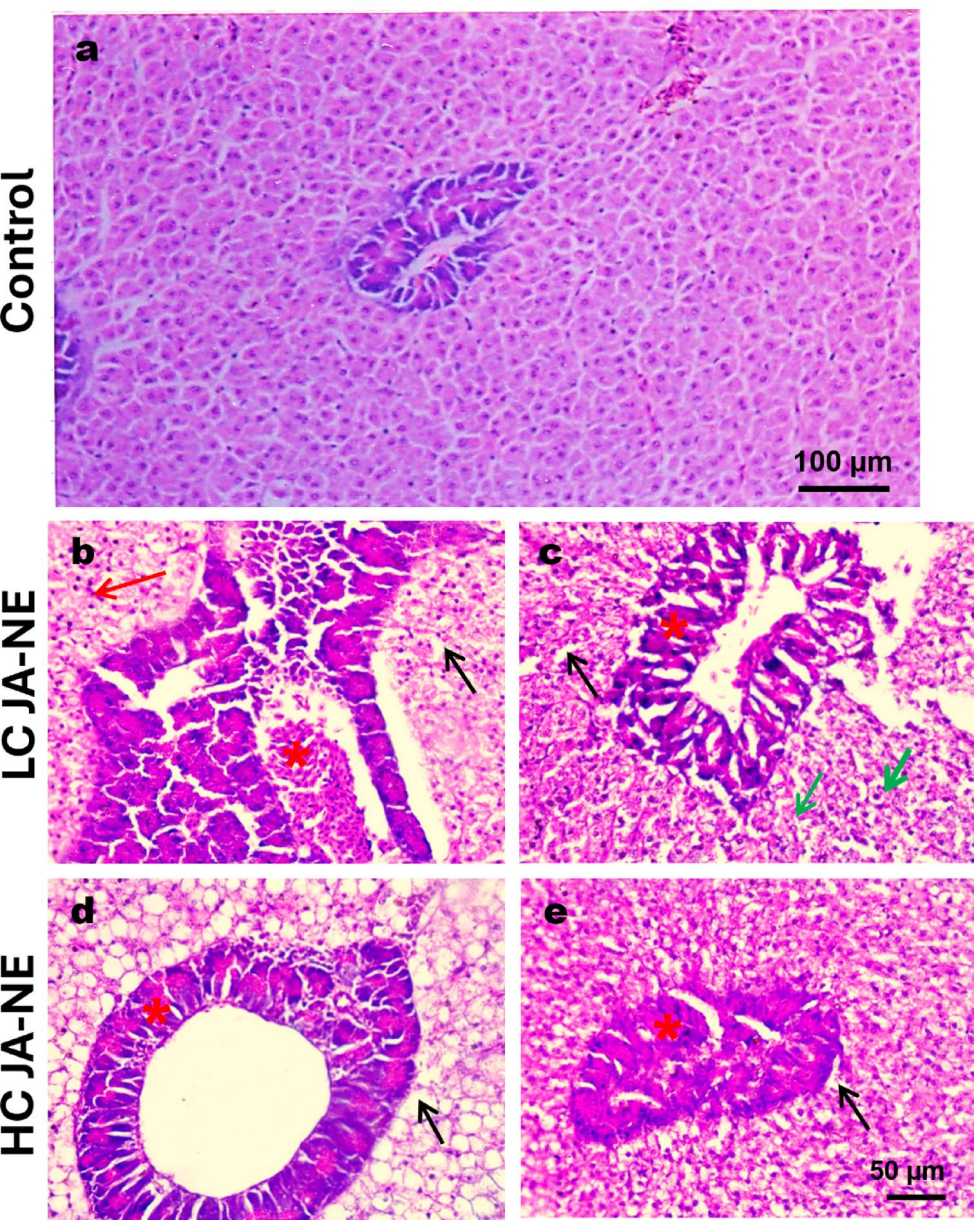
Photomicrograph of histological sections on the livers of Nile tilapia (*Oreochromis niloticus*). (**a**) control group was conducted without any substance. (**b**, **c**) the livers of *Oreochromis niloticus* exposed to low concentration (5%) of JA-NE. (**d**, **e**) the livers of *Oreochromis niloticus* exposed to a high concentration (10%) of JA-NE. Cytoplasmic vacuolization (black arrow), congestion of hepatic blood sinusoids (red arrow), necrobiotic changes in hepatopancreas with congestion (red asterisk), pyknotic nucleus (green arrow). Hematoxylin and Eosin stain, scale bar: **a**: 100 µm, **b**–**e**: 50 µm (X400). All panels were captured at the same magnification and with the scale bars.

**Fig. 5:**
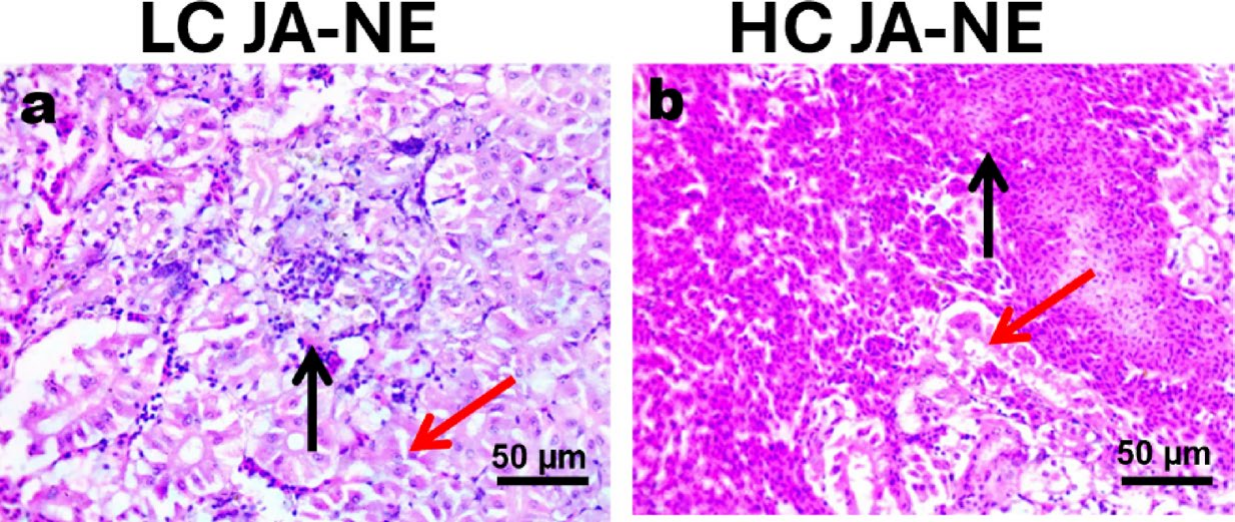
Photomicrograph of histological sections on the kidneys of Nile tilapia (*Oreochromis niloticus*). The kidneys of *Oreochromis niloticus* exposed to low concentration (5%) of JA-NE (**a**) and high concentration (10%) of JA-NE (**b**), showing peritubular hemorrhage and peritubular infiltration by mononuclear inflammatory cells (black arrow), some renal tubules showing necrobiotic changes (red arrow). Hematoxylin and Eosin stain, scale bar: 50 µm (X400).

**Fig. 6:**
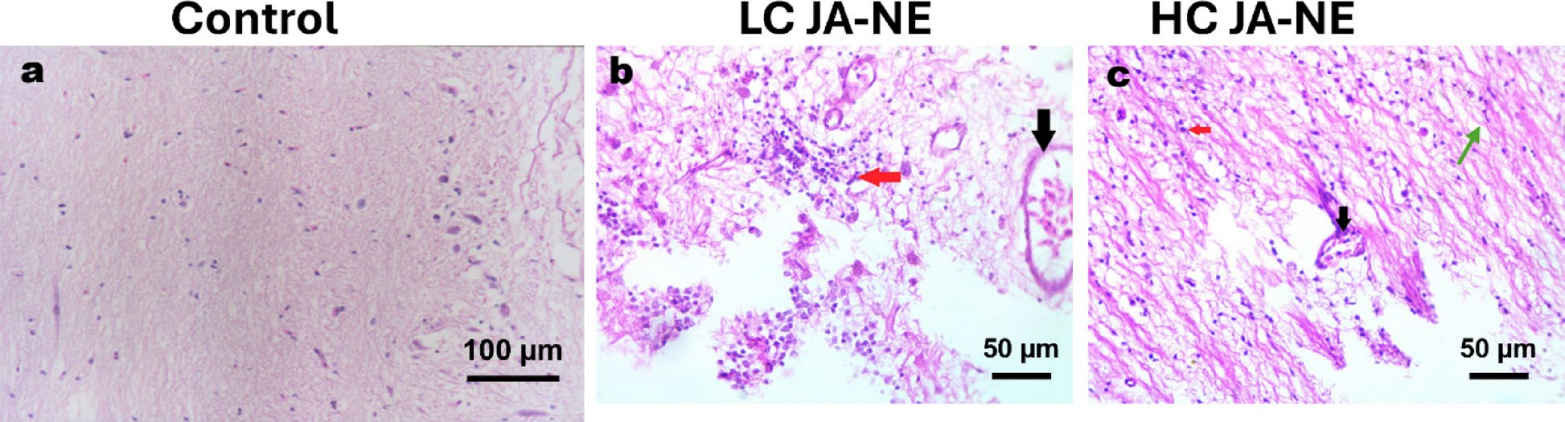
Photomicrograph of histological sections on the brains of Nile tilapia (*Oreochromis niloticus*). (**a**) control group was conducted without any substance. The brains of *Oreochromis niloticus* exposed to low concentration (5%) of JA-NE (**b**), and high concentration (10%) of JA-NE (**c**), showing congestion of minute blood capillaries in the brain (black arrow) with diffuse gliosis (red arrow), vacuolar degeneration in neurons (green arrow). Hematoxylin and Eosin stain, scale bar: **a**: 100 µm, **b**, **c**: 50 µm (X400).

Figures (3, 4, 5, 6) showed the histological results of the livers, kidneys, gills, and brains of Nile tilapia, *O. niloticus*, from high and low concentration groups.

#### Gills

Significant histological alterations in the gill architecture were observed in all exposed groups. The gills of LC of JA-NE exposed fish showed secondary gill lamellae fusion caused by epithelial cell hyperplasia (Fig. 3b, c). A mixture of pavement cells, mucous cells, chloride cells, and mononuclear inflammatory cells nearly entirely infiltrate interlamellar sulci. Additionally, telangiectasia was revealed in the secondary filaments. Meanwhile, fish exposed to HC of JA-NE exhibited gills with secondary lamellae lacking and hypotrophy, congestion of blood vessels and mucous cell diffusion (Fig. 3d, e).

#### Livers

The liver of the control group showed no discernible alterations (Fig. 4a). The liver revealed significant histological changes after 28 days of prolonged JA-NE treatment. A higher concentration of JA-NE was associated with more liver abnormalities (Fig. 4d & e). JA-NE treatment caused congestion of hepatic blood sinusoids and hepatopancreas necrosis. Pyknotic nuclei and contracted cytoplasm were features of necrotic cells. In addition, all exposed groups revealed vacuolar hepatocyte degradation. Some hepatocytes of LC of JA-NE showed vacuolar degeneration with pyknotic nuclei (Fig. 4b, c). However, HC of JA-NE presented severe vacuolar degeneration in all hepatocytes (Fig. 4d, e). Figure 4d shows congestion of minute blood capillaries in the hepatopancreas.

#### Kidney

Histopathological changes in kidney tissues were observed in all exposed fish, along with peritubular hemorrhage and peritubular infiltration by mononuclear inflammatory cells. Furthermore, necrobiotic changes were observed in several renal tubules (Fig. 5 a, b).

#### Brain

In the control group, no abnormalities or inconsistencies were observed in the brain tissue. However, following exposure to JA-NE, several pathological changes were noted. These included cerebral capillary congestion accompanied by focal gliosis, as illustrated in Fig. 6b, c. Additionally, vacuolar degeneration in neurons was identified, indicating potential cellular damage or stress in the brain tissue.

## Discussion

In recent years, essential oils (EOs) have been strongly recommended, since they are easily extracted and freely metabolized in the environment. The strong antibacterial and pesticidal potential of essential oils (EOs) makes them highly promising for use in agricultural and food preservation applications^46–48^.

Essential oil nano-emulsions are a novel formulation that combines the benefits of essential oils with the advantages of nanotechnology, which provides more efficacy, better stability, and higher bioavailability^10,49^. While increasing manufacturing and application of nano-emulsions, it’s crucial to consider their potential impact on aquatic ecosystems, particularly on fish^50^.

This study aimed to determine whether the Nile tilapia, *O. niloticus*, an economically significant fish species, experienced histological changes in gill, liver, kidney, and brain tissues during a short period of exposure to sublethal doses of JA-NE, as well as changes in biochemical indicators. Remarkably, no information regarding JA-NE’s toxicity to fish is accessible. To the best of our knowledge, this study represents the first investigation into the biochemical and histological changes in fish exposed to JA-NE. While jasmine EO is primarily known for its fragrance and therapeutic properties, recent studies have shown that it also possesses insecticidal properties^10,51^, antibacterial activity^52^ and cytotoxic potential^53^.

The LC_50_, which typically indicates the acute toxic effect, is widely used to determine how sensitive fish are to a certain chemical contaminant. The present study described that LC_50_ after 96 h of JA-NE was over 100 mg/L. These higher lethal doses indicate that JA-NE, like other EOs, is regarded as safe and ecofriendly chemicals^48^.

Neem oil nanoemulsions have shown superior antimicrobial activity compared to conventional formulations^54^, attributed to their small droplet size (160–507 nm), which enhances bioavailability and accelerates action against pests like *Sitophilus oryzae* and *Tribolium castaneum*. In aquatic models such as *Labeo rohita*, these nanoemulsions have also been associated with increased LC₅₀ values and changes in enzymatic biomarkers, suggesting altered toxicity profiles relative to bulk neem oil^55^. Thyme oil nanoemulsions (≤ 100 nm) were recently used as sedatives for transport of juvenile *Colossoma macropomum*, altering physiological biomarkers without causing mortality^56^.

Linalyl acetate, the main component identified in Jasmine oil^10^, exhibits significant anti-inflammatory and antioxidant properties^57,58^.

In aquatic animals, biochemical analysis can be utilized to assess overall health, immunological function, metabolic state, and disease tolerance^59,60^. The current work utilized ALT, AST, and ALP to assess liver function, while urea and creatinine levels have been used to represent renal function.

Surprisingly, our study revealed that fish exposed to 5% of JA-NE showed a significant increase in ALT/AST levels. This unexpected finding might be attributed to the hepatoprotective properties of linalool, a major constituent of JA-NE^57,61^. Other liver function parameters, such as ALT, ALP, TP, Alb, and Glb, showed minimal or non-significant changes, indicating that the liver’s synthetic and metabolic functions were largely unaffected.

Its antioxidant qualities and capacity to scavenge reactive oxygen species are also involved^57^. MDA is a crucial marker for determining how much ROS has accumulated in the body^62^. In the current study, exposure to JA-NE increased the concentrations of the oxidative stress biomarker (MDA) and antioxidant enzymes (GSH and GST). In fish, exposure to clove oil significantly elevated hepatic MDA levels and altered the activities of key antioxidant enzymes such as SOD, CAT, and GSH-Px, indicating lipid peroxidation and oxidative damage^63^. The JA-NE may induce free radicals leading to oxidative stress damage in tissues, which is why more antioxidant enzymes were made to lessen the negative effects of free radicals. The elevated MDA, GSH, and GST levels may be an indicator of lipid peroxidation or a reduction in antioxidant defense^64^. Similarly, in a related study, increased MDA levels accompanied by decreased GSH and GST activity in tilapia exposed to nanoparticles provided further evidence of oxidative damage and compromised antioxidant capacity^65^.

JA-NE exposure in Nile tilapia led to tissue damage in the gills, liver, kidneys, and brain, primarily due to oxidative stress. Elevated MDA levels indicated lipid peroxidation, while disruptions in GSH and GST activities reflected compromised antioxidant defenses. These molecular imbalances were linked to structural damage such as liver vacuolation, kidney necrosis, and gill fusion. Similar effects were reported with silver nanoparticle exposure, where oxidative damage and reduced antioxidant activity caused progressive liver injury^66^. Similarly, zinc oxide nanoparticle exposure in Nile tilapia caused marked reductions in muscle GSH, GST, and other antioxidant enzymes, reinforcing the mechanistic connection between oxidative stress and tissue-level damage^65^. Collectively, these findings highlight oxidative stress as a key driver of JA-NE-induced histopathological alterations in non-target fish species. Chamomile oil-alumina nanoformulations enhanced antioxidant defenses and reduced lipid peroxidation^67^, while basil oil nanoemulsions improved disease resistance and modulated immune and antioxidant responses^68^. These findings align with our results and highlight oxidative stress as a central mechanism in nanoemulsion-induced toxicity.

The health and immunological state of fish are closely linked to their antioxidant defense mechanism. Cytokines play a crucial role in modulating immune responses, and fish often rely on immune-regulatory genes that generate proinflammatory cytokines, including IL-1β, TNF-α, and IL-6^69,70^.

Our results indicate that a low concentration of JA-NE (5%) can reduce the TNF-α level due to its anti-inflammatory effect^58^. In line with this, Jasminum extracts can diminish levels of IL-1β, TNF-α, and IL-6 in colitic rats when they were administered with 400 mg/kg for 7 days^71^. The elevated levels of pro-inflammatory cytokines IL-1β and TNF-α observed in Nile tilapia following JA-NE exposure likely indicate activation of innate immune pathways, particularly the NF-κB and p38 MAPK signaling cascades. In fish, stimulation of toll-like receptors (TLRs) by chemical or pathogenic stressors initiates MyD88-dependent pathways, leading to activation of IKK and p38 MAPK, followed by nuclear translocation of NF-κB (p65), which promotes transcription of IL-1β and possibly TNF-α^72^. JA-NE exposure may trigger oxidative stress and activate TLR-mediated pathways, leading to pro-inflammatory cytokine expression through NF-κB and MAPK signaling. This supports the observed immune responses and aligns with known immunotoxicological mechanisms. Future research should explore pathway activation and marker expression to clarify JA-NE’s immune and neuro-endocrine effects in fish.

The effects of different toxicants on fish are indicated by histological investigation^73^. The current study revealed that there were some alterations in gill, liver, kidney, and brain tissues after exposure to JA-NE. Gills of JA-NE exposure fish showed lamellae fusion. Lamellar fusion happens when hyperplasia keeps growing and new cells infiltrate the gaps between secondary lamellae, which in turn creates adhesions on both sides of the lamella^74^. Our results align with a study by^75^ on *Mentha piperita* EO toxicity in *Arapaima gigas*, which reported gill alterations, including fused lamella, epithelial elevations, aneurysm, and hypertrophy. Notably, necrosis was exhibited at high concentrations of *M. piperita*. Over-proliferation of gill cells is thought to be caused by heavy metals, ammonia poisoning, change of water pH, and parasitic infections^76,77^.

One of the organs most susceptible to any chemical is the liver^19,78^, and therefore it can be utilized as an indicator for environmental monitoring. Thus, changes in its structure can therefore be used to evaluate fish health^79^. In the present study, fish exposed to both HC and LC of JA-NE, separately, displayed different pathological changes in their liver tissue, including vacuolization, necrosis, blood congestion, hemorrhage, and hypertrophic nucleus.

Numerous EO research studies have also assessed the oil’s potential for toxicity using biochemical, hematological, and histopathological markers on fish^80,81^.

Based on earlier studies, fish kidneys are the major organs exposed to various contaminants, such as pesticides, insecticides, and herbicides in water^82^. Jasmine oil is a complex mixture of various chemical compounds, primarily terpenoids like linalool, benzyl acetate, and benzyl alcohol^83,84^. These compounds, while often considered safe for humans in small amounts, can be toxic to aquatic organisms at certain concentrations. Terpenoids can directly damage kidney tubules, which disrupt vital functions like filtration and reabsorption, leading to kidney dysfunction^85^.

Since creatinine is less affected by other variables like nutrition and hydration, it is a more accurate measure of renal function. Results in Fig. 1 provide the increased levels of creatinine in both concentrations of exposed fish. A high ratio of creatinine indicates pre-renal disorders. These results are consistent with a prior study into the toxicity of lemon balm (*Melissa officinalis*) to rats^86^, which demonstrated that lemon, which consists of terpenes, can elevate creatinine levels in rats, suggesting acute kidney damage. Additionally, other factors can raise its level, such as hyperthyroidism, infections, burns, fractures, and skeletal muscular atrophy or necrosis^87^.

Capillary congestion in the fish brain suggests impaired blood flow, which can lead to hypoxia (oxygen deficiency) and nutrient deprivation in brain tissues. This phenomenon is often associated with vascular stress or inflammation caused by toxic substances^88^. The congestion observed here may indicate that JA-NE disrupts normal vascular function in the brain. Focal gliosis indicates an activation of glial cells (the brain’s support cells) in response to damage or stress. This is typically a reactive process to injury, inflammation, or toxicity, suggesting that JA-NE may have induced localized brain damage^89^. Vacuolar degeneration in neurons is a sign of cellular damage, where vacuoles (empty spaces) form within the neurons. This is often associated with cellular stress, toxicity, or degenerative processes, implying that JA-NE may have neurotoxic effects^43^. The observed structural alterations in the kidney and brain of Nile tilapia may reflect potential endocrine disruption or neurotoxicity associated with the terpene-rich composition of JA-NE (such as linalool and benzyl acetate). Similar adverse effects have been reported in fish exposed to herbicides and related xenobiotics; for example, acute exposure to pendimethalin induced oxidative stress, disrupted globulin levels, and caused metabolic disturbances in *O. niloticus*^90^. Moreover, exposure to the phenylurea herbicide tebuthiuron has been shown to induce notable hormonal imbalances in male tilapia, characterized by reduced testosterone levels, elevated estradiol concentrations, and associated testicular degeneration^91^. These findings are consistent with our observations of renal and neural histopathological damage, suggesting that JA-NE owing to its terpene-rich composition may trigger endocrine-disrupting or neurotoxic pathways in non-target fish. To clarify these potential mechanisms, future research should focus on evaluating specific endocrine biomarkers and neurotransmitter profiles.

This study emphasizes the significance of taking a comprehensive approach to pest management that strikes a balance between ecological integrity and efficacy by bridging the gap between innovation and environmental safety. Accurate prior knowledge of the Eos’ safety margin, concentrations, and exposure duration is necessary to ensure the exposed fish’s survival^75,80,81^.

## Conclusions

This study provides critical insights into the biochemical and histopathological effects of Jasmine oil nanoemulsion (JA-NE) on Nile tilapia (*Oreochromis niloticus*), highlighting its dual role as both a promising eco-friendly agent and a potential stressor to aquatic organisms. While JA-NE demonstrated low acute toxicity, with no mortality observed at concentrations up to 100 mg/L (100%), sub-lethal exposure at 5% and 10% over 28 days induced significant alterations in hepatic, renal, and immune functions and resulted in oxidative stress, immune modulation, and histopathological damage in gills, liver, kidneys, and brain. Elevated levels of oxidative stress indicators and histopathological damage in gills, liver, kidneys, and brain tissues underscore the need for cautious application of JA-NE in aquatic environments. To enhance practical applicability, we propose that concentrations below 5% may represent a preliminary safety threshold for short- to medium-term exposure in aquaculture settings, though further chronic toxicity studies are recommended. Stakeholders are encouraged to implement mitigation strategies such as water quality monitoring and controlled release systems to minimize unintended ecological impacts. Future research should prioritize long-term ecotoxicity profiling, recovery potential post-exposure, and formulation refinements that enhance safety without compromising efficacy. Additionally, semi-quantitative histopathological scoring should be integrated to strengthen the assessment of tissue-level alterations and allow for more robust dose–response correlations. This will improve cross-study comparability and provide clearer guidance on safe exposure limits.

The findings emphasize the importance of balancing the benefits of essential oil nanoemulsions, such as their antibacterial and pesticidal properties, with their potential ecological risks. This study supports the Sustainable Development Goals (SDGs) of the United Nations, especially those pertaining to sustainable aquaculture (SDG 14) and responsible consumption and production (SDG 12), by advocating for environmentally safe pest management strategies^92^. This study contributes to a deeper understanding of environmental interaction between nanoemulsions of botanical insecticides and aquatic organisms, paving the way for the development of safer and more sustainable approaches for mosquito control. Overall, JA-NE represents a promising tool for sustainable agriculture and aquaculture, provided its application is carefully regulated to safeguard aquatic ecosystems.

## Data Availability

All data generated or analyzed during this study are included in this published article.
